# Periodontal treatment effects on endothelial function and cardiovascular disease biomarkers in subjects with chronic periodontitis: protocol for a randomized clinical trial

**DOI:** 10.1186/1745-6215-12-46

**Published:** 2011-02-16

**Authors:** Jorge H Ramírez, Roger M Arce, Adolfo Contreras

**Affiliations:** 1 Periodontal Medicine Research Group, Department of Periodontology, School of Dentistry, Universidad del Valle, Calle 4B 36-00, Cali, Colombia; 2 Medical School Director, Faculty of Health Sciences, Universidad Icesi, Calle 18 122-135, Cali, Colombia; 3 Center for Oral and Systemic Diseases, NC Oral Health Institute, School of Dentistry, University of North Carolina at Chapel Hill, Chapel Hill, NC, USA

## Abstract

**Background:**

Periodontal disease (PD) is an infectious clinical entity characterized by the destruction of supporting tissues of the teeth as the result of a chronic inflammatory response in a susceptible host. It has been proposed that PD as subclinical infection may contribute to the etiology and to the pathogenesis of several systemic diseases including Atherosclerosis. A number of epidemiological studies link periodontal disease/edentulism as independent risk factor for acute myocardial infarction, peripheral vascular disease, and cerebrovascular disease. Moreover, new randomized controlled clinical trials have shown an improvement on cardiovascular surrogate markers (endothelial function, sICAM, hsPCR level, fibrinogen) after periodontal treatment. Nonetheless, such trials are still limited in terms of external validity, periodontal treatment strategies, CONSORT-based design and results consistency/extrapolation. The current study is designed to evaluate if periodontal treatment with scaling and root planning plus local delivered chlorhexidine improves endothelial function and other biomarkers of cardiovascular disease in subjects with moderate to severe periodontitis.

**Methods/Design:**

This randomized, single-blind clinical trial will be performed at two health centers and will include two periodontal treatment strategies. After medical/periodontal screening, a baseline endothelium-dependent brachial artery flow-mediated dilatation (FMD) and other systemic surrogate markers will be obtained from all recruited subjects. Patients then will be randomized to receive either supragingival/subgingival plaque cleaning and calculus removal plus chlorhexidine (treatment group) or supragingival plaque removal only (control group). A second and third FMD will be obtained after 24 hours and 12 weeks in both treatment arms. Each group will consist of 49 patients (n = 98) and all patients will be followed-up for secondary outcomes and will be monitored through a coordinating center. The primary outcomes are FMD differences baseline, 24 hours and 3 months after treatment. The secondary outcomes are differences in C-reactive protein (hs-CRP), glucose serum levels, blood lipid profile, and HOMA index.

**Discussion:**

This RCT is expected to provide more evidence on the effects of different periodontal treatment modalities on FMD values, as well as to correlate such findings with different surrogate markers of systemic inflammation with cardiovascular effects.

**Trial registration number:**

ClinicalTrials.gov Identifier: NCT00681564.

## Background

Cardiovascular disease continues to be the main cause of morbidity and mortality worldwide. Despite the existence of novel therapeutic approaches designed for the prevention and treatment of atherosclerosis, the number of deaths associated to cardiovascular events remains constant in most countries[1]. For instance, in Colombia, one out of five deaths can be attributed to ischemic cardiovascular disease[2]. During the last decade it has been widely accepted that inflammation plays a key role in the development of atherosclerosis. Multiple epidemiological studies have confirmed the association between high levels of acute phase reactants such as C-reactive protein (CRP), fibrinogen, Serum Amyloid A and soluble adhesion molecules like ICAM-1, E-Selectin, VCAM-1 with the progression of atherosclerosis and also with an increased risk for cardiovascular disease[3].

New scientific evidence from the last two decades including epidemiological, *in vivo *and *in vitro *assays supports the notion that the immune system significantly contributes in the development and progression of atherosclerosis[4]. This new theory proposes that any potential noxious challenge to the host immune response could be related to the pathogenesis of atherosclerosis[5]. Hence, other nontraditional risk factors for cardiovascular events, such as infections and rheumatologic autoimmune diseases have emerged as important risk factors[6].

Several epidemiological studies have also suggested that periodontal infection is an independent risk factor for acute myocardial infarction, peripheral vascular disease and cerebrovascular disease. A recent meta-analysis on the subset of five cohort studies (86,092 patients, follow-up > 6 years) found increased incidence of coronary heart disease (RR = 1.24, 95% CI 1.14-1.36, p < .0001) in patients with less than 10 teeth. In the subset of cross-sectional studies at the same meta-analysis report, prevalence of coronary heart disease was reported to be significantly high (OR = 1.59, 95% CI 1.329-1.907, p < .001)[7]. The association between periodontitis and cardiovascular disease in meta-analysis literature is stronger when systemic inflammatory and serologic markers are used to determine the systemic bacterial exposure secondary to periodontitis[8].

Several biological mechanisms have been suggested to explain the association between periodontal infections and atherosclerosis:

### 1. Systemic consequences of periodontal infection (indirect pathway)

Patients with periodontitis have increased levels of C-reactive protein, fibrinogen, TNF-α, IL-1, IL-6 and other acute phase reactants associated to cardiovascular events[9,10]. Proinflammatory cytokines (TNF-α, IL-1, IL-6) reduce the expression of endothelial Nitric Oxide Synthase (eNOS),[11,12] increase endothelial synthesis of NADPH oxidase,[13] and promotes the expression of endothelial cell adhesion molecules (e-Selectin, ICAM-1, VCAM-1)[14]. It is well-know that the absence of anti-atherogenic properties in the endothelium augments the vascular migration of leukocytes (diapedesis) to atherosclerotic plaques[4]. In addition, increased activation of platelets has been reported in subjects with periodontitis[15]. Despite the evidence, the contribution to other classic risk factors of CVD of the systemic inflammation associated with periodontitis remains largely unknown.

### 2. Invasion of periodontal pathogens into atherosclerotic plaques (Direct pathway)

Periodontal pathogens (*i.e., Porphyromonas gingivalis, Aggregatibacter actinomycetencomitans, Prevotella intermedia, Treponema denticola*, and *Eikenella corrodens*) have been found in atherosclerotic plaques[16,17]. Recent studies have shown that invasion by *P. gingivalis *induces the expression of endothelial cell adhesion molecules, IL-8, IL-6, MCP-1, and TLR-4[18-20]. *P. gingivalis *HSP60 (GroEL) induces TLR-2 and TLR-4 expression on the surface of endothelial cells,[21] suggesting that autoimmune mechanisms secondary to periodontal infections could play a role in the progression and development of atherosclerosis[22].

Randomized controlled trials (RCTs) are therefore important to study the impact of periodontal infections on cardiovascular disease. A growing number of clinical trials designed to study the effects of periodontal treatment on cardiovascular disease have been published during the last decade[23-27]. All RCTs evaluating conventional periodontal therapy effects have used different biomarkers of inflammation and endothelial function as surrogate outcomes for cardiovascular events, whereas definitive endpoints such as death, ischemic heart disease and cerebrovascular accident are not frequently used. For instance, clinical trials with definitive cardiovascular endpoints in periodontitis patients are not available due to methodological limitations (*i.e. *larger sample sizes, or long term follow-up) and/or ethical aspects concerning the non-treatment of subjects with periodontitis for long periods of time.

Hence, the use of surrogate biomarkers in RCT evaluating the effects of periodontal treatment in the prevention of cardiovascular disease is justified, because the otherwise more definitive outcomes such as cardiovascular events, cardiovascular deaths, and hospitalizations for cardiovascular complications requires long follow-up periods. Surrogate biomarkers such as flow-mediated, endothelium dependent vasodilatation of the brachial artery (FMD) is a non-invasive, reproducible and easy technique for measuring endothelial function in humans[28]. FMD is decreased in subjects with cardiovascular risk factors (i.e. diabetes, hypertension, obesity, and smoking among others) and atherosclerosis[29]. Furthermore, positive changes in lifestyle (diet, smoking cessation and physical exercise) as well as cardiovascular drugs (ACE inhibitors, statins, oral hypoglycemic drugs, and calcium antagonists) improve endothelial function in humans[30-32]. The use of FMD as a biomarker for cardiovascular disease has been recently supported by studies reporting association between endothelial dysfunction and cardiovascular events[33].

To date, there are some published RCTs that have found an improvement on endothelial function after periodontal treatment (REF). However, there are several reasons to justify the development of more clinical trials to study this association:

1. Extrapolation of results to the general community (external validity): Only subjects with chronic severe generalized periodontitis (less than 1% of the adult population) have been included in clinical trials of periodontal treatment and endothelial function,[23-27] therefore there is no available evidence that periodontal treatment improves endothelial function in subjects affected by slight or moderate periodontitis, both more prevalent forms of periodontal disease. Furthermore, the effects of periodontal treatment on endothelial function in diverse ethnic/social groups with other co-morbidities (*i.e. *obesity, diabetes, cardiovascular disease, metabolic syndrome, chronic renal failure) are unknown.

2. Differences in periodontal disease treatment strategies: some treatment protocols of periodontal disease used in some RCTs are not available or affordable in non-industrialized countries (*i.e. *local antibiotic therapy with minocycline microspheres).

3. Methodological limitations of published trials: most published trials do not include a control group (single arm clinical trials) and only one RCT published to date by Tonnetti et al.[25] fully complied with the CONSORT statement[34]. Moreover, information regarding the periodontal diagnosis and disease parameters of the subjects included in recent clinical trials is frequently incomplete.

4. Consistency: results are consistent when the association persists despite the use of alternative designs, by different researchers and different geographic locations. More RCTs with design improvements and conducted by different research groups are required to confirm the beneficial effects of periodontal treatment on biomarkers of cardiovascular disease.

In summary, current evidence supports a biologically plausible association between periodontal disease and atherosclerosis. To date, few studies have evaluated the effects of periodontal treatment on endothelium-dependent brachial artery flow-mediated dilatation. Moreover, the evidence obtained from observational studies is still controversial, probably due to selection biases that have resulted in weaker association strengths. This RCT will be conducted to help to confirm a possible association between periodontitis and cardiovascular disease, to unveil some biological mechanisms by which periodontal treatment could reduce cardiovascular risk and to increase the external validity of previous studies.

The study hypothesis is that periodontal treatment with scaling and root planning supplemented with chlorhexidine (Full-Mouth Disinfection) - without systemic antibiotics, improves endothelial function and other biomarkers of cardiovascular disease in subjects with moderate to severe periodontitis. **ClinicalTrials.gov Identifier: NCT00681564**.

## Methods/Study Design

### Elegibility

#### - Enrolling of study participants

The periodontal status of potential study participants will be examined by a single periodontists at recruitment (JQ) who will not be involved in any treatment or follow-up of research subjects. A physical examination and structured interview by a certified physician (JR) will be performed to assess eligibility. A member of the research team will explain the study protocol and the informed consent to all the eligible subjects. Inclusion and exclusion criteria are depicted in Table 1.

**Table 1:** Inclusion and exclusion criteria

Inclusion criteria
• Male or female
• 25 years of age or older
• Three or more periodontal pockets with a probing depth (PD) > 5 mm
• Have at least 16 natural teeth excluding third molars
• Provide informed consent and willingness to cooperate with the study protocol

**Exclusion Criteria**

• History of antibiotic use in the previous three months
• Pregnant or lactating females
• Treatment with antihypertensive, antilipemic, antiarrhythmic, and other cardiovascular drugs
• Systemic diseases such as diabetes, HIV/AIDS, liver disease, chronic renal failure, tuberculosis, and autoimmune diseases
• Previous history of cardiovascular disease: Acute myocardial infarct, stable angina, unstable angina, heart failure, atrial fibrillation, AV blockade, peripheral vascular disease, and cerebrovascular accident
• Patients who received periodontal treatment within the last 6 months
• Patients who require antibiotic prophylaxis before examination or treatment
• Patients with some mental disability

### Settings

Treatments and tests (blood samples, endothelium-dependent brachial artery flow-mediated dilatation, radiographs, subgingival samples and microbial cultures) of this RCT will be performed at the Universidad del Valle at Cali, Colombia. Patients will be recruited from *State-funded primary care Health Centers *(Cañaveralejo and Siloe), and Universidad del Valle dental school, located in the same geographical region of Cali.

### Study design

**Figure 1:**
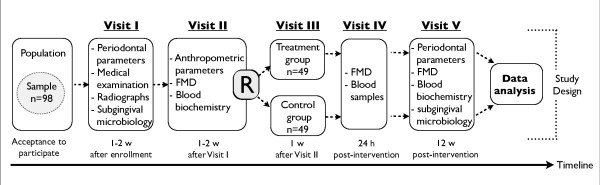


This is a single blind (evaluators of study variables and outcomes) clinical trial designed to evaluate the effects of periodontal treatment on endothelium-dependent brachial artery flow-mediated dilatation in 98 subjects with moderate to severe periodontitis. Eligible subjects for the present study will be invited to the following visits (Figure 1):

#### - Visit I

periodontal clinical parameters, subgingival samples collection (for microbial cultures and Polymerase Chain Reactions to detect specific periodontal pathogens), periapical radiographs, medical examination, and structured interview at the School of Dentistry (Universidad del Valle).

#### - Visit II

baseline endothelium-dependent brachial artery flow-mediated dilatation and anthropometric parameters (weight, height, and abdominal circumference). A fasting blood sample will be obtained to determine glucose, insulin, hs-CRP, lipid profile, and a pro-inflammatory cytokine panel by Multiplex^®^.

#### - Visit III

randomization to supragingival and subgingival plaque cleaning and calculus removal, four quadrants in one session, plus chlorhexidine (treatment group, full-mouth disinfection protocol) or supragingival plaque removal (control group, adult prophylaxis). All the interventions will be performed by two qualified periodontists at the School of Dentistry (Universidad del Valle), at Cañaveralejo Health Center or at Siloe Health Center.

#### - Visit IV (24 hours after periodontal treatment)

endothelium-dependent brachial artery flow-mediated dilatation and fasting blood samples at the School of Basic Sciences (Universidad del Valle).

#### - Visit V (12 weeks after periodontal treatment)

endothelium-dependent brachial artery flow-mediated dilatation, periodontal parameters, and subgingival samples at the School of Basic Sciences at the Universidad del Valle. A fasting blood sample will be obtained to determine glucose, insulin, hs-CRP, lipid profile, and cytokines by Multiplex^®^.

#### - Visit VI

follow-up (intervention group) and treatment (control group) performed by a periodontist at the School of Dentistry (Universidad del Valle), Cañaveralejo or Siloe.

### Outcomes

#### - Primary outcome

The primary outcome is the difference on endothelium-dependent brachial artery flow-mediated dilatation (FMD) at baseline and three months after randomization [Time Frame: Baseline, 24 h, and 12 weeks after treatment].

#### - Secondary outcomes

Secondary outcomes of this study are hs-CRP and glucose serum levels, blood lipid profile, and HOMA index [Time Frame: Baseline, 24 h and 12 weeks after treatment].

### Interventions

All procedures in the control and treatment group will be performed by three certified periodontists following standard treatment guidelines. Standard Operating Procedures (SOPs) will be developed for the standardization of the interventions in the control and treatment groups. To ensure the uniform delivery of the interventions, an experienced periodontist (JS, Director of Periodontology Program at Universidad del Valle) will be responsible for the training of the team of dental professionals involved in the delivery of the interventions. In addition, a videotape of the interventions were obtained and reviewed to fully comply with the protocol. Patients randomized to the *control group *received a conventional adult prophylaxis (dental cleaning), consisting in one session of supragingival removal of plaque biofilm with the use of mechanical scaling and coronal polishing. Patients randomized to the *treatment group *will be treated using the one-stage full-mouth disinfection protocol consisting in the following procedures:

• Administration of local anesthesia [lidocaine (1%) with epinephrine]

• Scaling and root planing with ultrasonic devices and curettes, four quadrants in one session

• Tongue brushing with a 1% chlorhexidine gel (1 minute)

• Mouth rinsing with a 0.2% chlorhexidine solution for (2 minutes)

• Subgingival chlorhexidine (1%) irrigation in all pockets

• Twice daily rinsing with chlorhexidine (1 minute) during fourteen days after the periodontal intervention

• Dental extractions will be performed at the end of patient follow-up (only in cases of teeth that could not be preserved)

### Sample size

The primary outcome FMD was selected as the critical variable to calculate the sample size. 49 patients (98 in total) in each group are required to have an 80% chance of detecting as significant (at the two sided 5% level) a 1% difference between the two groups in the mean FMD, with assumed standard deviation of 2.6 and a planned 15% rate of missing patients on the follow up.

### Randomization, allocation concealment and blinding

Subjects will be randomized into one of two groups: 1) treatment group (one stage full-mouth disinfection), and 2) control group (conventional adult prophylaxis). Randomization will be stratified on the basis of gender (male or female), and smoking status (*current smokers*: subjects who reported regular smoking of one or more cigarettes a day for at least one year; *non-smokers*: never smoke and former smokers who had stopped smoking for at least 12 months). The sequence will be generated using a random number generator in a computer by an independent researcher not involved in the interventions or measuring the clinical variables. Random permuted blocks with a size of four patients will be selected. Sequentially numbered, opaque, sealed envelopes (SNOSE) were developed for the implementation of the random sequence.

On the treatment day, a trained nurse will open the corresponding numbered envelope at the Cañaveralejo Health Center or at the dental clinics Universidad del Valle. The patient's group allocation will be given to the periodontist to perform the corresponding intervention. Due to the nature of the interventions, it is no possible to blind study participants and periodontists for the treatment protocol. Outcome evaluators and data analysts will be blinded to the group assignment. Detailed instructions on methods to maintain the blind will be given to the researchers, health care providers and outcome evaluators. Furthermore, these instructions were reinforced in bimonthly meetings with the personal involved in the study and detailed in the operations manual.

### Periodontal examination and subgingival samples

Calibrated dentists with experience in performing periodontal examinations will perform the evaluation of the periodontal status of recruited patients. The following clinical parameters will be evaluated:

A. Number of present teeth.

B. Gingival Margin Level (GML): distance in millimeters from the cemental-enamel junction (CEJ) to the gingival margin.

C. Probing Pocket Depth (PD): Distance in millimeters from the gingival margin to the periodontal pocket as measured with a calibrated periodontal probe.

D. Clinical Attachment Level (CAL): distance in millimeters from the CEJ to the deepest aspect of the periodontal sulcus or pocket.

E. Bleeding on Probing (BOP): the presence of BOP will be expressed as yes/no at each site. The extent of BOP will be reported as a percentage of all sites.

GML, PD, and CAL will be recorded at six sites per tooth. PD and CAL will be reported in each patient as follows:

A. Mean PD

B. Mean CAL

C. Number of sites with PD ≥ 4 and PD ≥ 5

D. Number of sites with CAL ≥ 3 and CAL ≥ 4

The six deepest pockets (>4 mm) on each patient were selected for subgingival microbial samples. The supragingival plaque will be removed with cotton gauze and a sterile curette. Subsequently, two absorbent paper points (#45) are introduced to the bottom of each pocket for a period of 20 seconds. Six points will be collected in a sterile Eppendorf tube and stored at - 70°C until analysis by Polymerase Chain Reaction (PCR). The remaining six points will be placed into vials containing transport medium VMGA-III for bacterial culturing and microbial colony identification.

### Blood samples

Venous blood samples will be withdrawn from the antecubital vein in fasting conditions (8 to 10 hours). Blood samples will be collected using three vacutainer tubes, one dry, one with EDTA and another with sodium heparin. One 1000 uL aliquote of the EDTA blood sample will be used for Complete Blood Count (CBC). All remaining samples will be centrifuged at 1500 rpm during 10 minutes to extract the plasma and serum. Plasma obtained during visits II, IV and V will be stored in Eppendorf vials at -70°C until the end of the study for the analysis of cytokines by Multiplex^®^, hs-CRP and insulin. Serum samples will be immediately analyzed for measurement of glucose and lipid profile.

### Biochemical markers

Biochemical tests will be performed in the Microbiology and Immunology Laboratory, from the Universidad del Valle (Cali, Colombia) and other private certified Clinical Laboratory.

*Plasma glucose levels*: glucose measurement will be performed using enzymatic-colorimetric techniques (Selectra, Merck).

*Lipid profile*: High-density lipoproteins cholesterol (HDL-C), total cholesterol (TC) and triglycerides will be determined by enzymatic-colorimetric techniques (Selectra, Merck). Low-density lipoproteins cholesterol (LDL-C) will be calculated using the Friedwald equation: LDL-C = TC-(HDL-C+TG/2.2).

*High sensitivity C-Reactive Protein (hs-CRP)*: the fasting plasma hs-CRP concentrations will be analyzed using a solid-phase, chemiluminescent immunometric assay (Immulite 1000, Siemens).

*Complete Blood Count (CBC)*: CBC will be performed using an automated analyzer (Advia 60, Siemens).

### Luminex multiplex assay for measurement of cardiovascular risk biomarkers

Cardiovascular risk biomarkers will be determined on plasma samples using the Luminex-200 system (Luminex Corporation). This system allows measuring up to 200 proteins simultaneously in a small sample volume (25-50 μL). MILLIPLEX™ MAP biomarker panel will be used to determine the following cardiovascular risk biomarkers: MMP-9, MPO, PAI-1 (total), sE-Selectin, sICAM-1, sVCAM-1, hs-CRP, fibrinogen, haptoglobine, amyloid A and amyloid P.

### Culture of periodontopathic and superinfecting bacteria

The samples will be analyzed using microbial culture techniques for the presence of periodontopathic bacteria according to Slots[35]. Briefly, all samples will be processed before 24 hours at room atmosphere (25°C) and immediately incubated in CO_2 _and anaerobic culture systems. Brucella blood agar medium will be incubated at 35°C in an anaerobic jar for 7 days. The TSBV medium will be incubated in 10% CO_2 _in air at 37°C for 4 days. Presumptive identification will be performed according to methods described by Slots & Reynolds and Slots et al.[36,37] to identify by colony morphology and Gram stain for *Campylobacter *spp., *Eubacterium *spp., *Fusobacterium *spp., *Capnocytophaga *spp., *Dialister pneumosintes*. Colony morphology characterized by the presence of an inner star, and catalase positive test will be used to identify *Aggregatibacter actinomycetemcomitans*. In addition, *Porphyromonas gingivalis, Prevotella intermedia/nigrescens, Tannerella forsythia, Micromonas micros *and *Eikenella corrodens *will be identified by the use of a commercial micromethod system (RapID ANA II, Remel, Norcross, GA, USA). Gram-negative enteric rods will be sub-cultured and colony purified on MacConkey and Cetrimide agar plates and identified using a standardized biochemical test (API 20E^®^, bioMerieux, Inc, Marcy l'Etoile, France). Total viable counts (TVC) will be defined as the total number of colony forming units obtained on non-selective media plates. Species found on selective media will be enumerated and presented as percentage (%) and counts × 10^5^. For the isolation of enteric bacteria, MacConkey agar will be used and incubated in aerobic atmosphere at 37°C for 24-48 hours. A Gram stain will be performed to colonies that grew on MacConkey agar medium for verification. The subgingival presence of Human Cytomegalovirus (HCMV) also will be determined at these samples using a nested PCR technique.

### Polymerase chain reaction (PCR)

Bacterial and Viral DNA extraction will be performed according to Boom et al.[38] Bacterial PCR will be performed as described by Ashimoto et al.[39] in and Saiki et al.[40] For *P. gingivalis*, *T. forsythia*, *E. corrodens *and *C. rectus *a 50 μl of a reaction mixture containing 10.0 μl sample template, 5.0 μl of 10× PCR buffer (50 mM KCl, 10 mM Tris-HCl [pH 9.0 a 25°C], 1.5 mM MgCl_2 _and 0.1% of Triton^® ^X-100) o PROMEGA0.25 U of Taq DNA polymerase, 5.0 μl (0.2 Mm) of each of deoxyribonucleotides, 1.0 μl (2 μM) of the primer from the bacteria being investigated and 3.0 μl MgCl_2 _(1.5 mM), was used. For *A. actinomycetemcomitans*, *P. intermedia *y *P. nigrescens *the concentration of MgCl_2 _was 2.25 mM

The primers used will be those recommended by Ashimoto et al.,[39] positive and negative controls will be included for each bacterium, and a 1 Kb molecular weight marker. The sample will be replaced by 10.0 μl sterile distilled water in the negative control and 10.0 μl of DNA from the bacteria being investigated will be included in the positive control.

Samples will be amplified in a thermal cycler (MyCycler™ Termal Cycler, Bio-Rad). The PCR temperature profile for *P. gingivalis*, *T. forsythia*, *E. corrodens *and *C. rectus *included an initial denaturation step at 95°C for 2 min followed by 36 cycles of: denaturation step at 95°C for 30 s, a primer annealing step at 60°C for 1 min, an extension step at 72°C for 1 min and a final step of 72°C for 2 min. The temperature profile for *A. actinomycetemcomitans*, *P. intermedia *y *P. nigrescens *will included an initial step of 95°C for 2 min followed by 36 cycles of: 94°C for 30 s, 55°C for 1 min, 72°C for 2 min and a final step of 72°C for 10 min.

*P. gingivalis *ATCC 33277, *C. rectus *ATCC 33238, *T. forsythia *ATCC 43037, E. corrodens ATCC 23834, *A. actinomycetemcomitans *ATCC 29522, *P. intermedia *ATCC 25611 y *P. nigrescens *ATCC 33563 reference strains will be used. *P. gingivalis *will be identified by the presence of an amplified product in the 404 base pair (bp) band, *C. rectus *by an amplified product in the 598 bp band, *T. forsythia *in the 641 bp band, *E. corrodens *in the 688 bp band, *P. intermedia *in the 575 bp band and *P. nigrescens *in the 804 bp band. The primers used will be the ones described by Ashimoto et al.[39] were selected with the help of the Ribosomal database Project (RDP) program.

A nested PCR for HCMV will be used to determine the subgingival viral presence. HCMV PCR conditions and primers will be selected as published by Parra et al.[41] Positive control - DNA extracted from HCMV Towne strain and negative control will be used during the experiments. A band of 123 base pair will represent a HCMV positive isolation.

Bacterial and viral PCR products will be electrophoretically fractioned at 4 V/cm in 1.5% agarose gel in TAE buffer (Tris acetate-EDTA) stained with cyber green and visualised on a transilluminator under 300 nm ultraviolet light.

The subgingival microbiota composition of the patients will be determined at baseline and at three months after study inclusion and will be analyzed as other independent microbial risk factor. The effect of periodontal treatment on subgingival microbiota composition will be also determined.

### Physical and anthropometric measurements

All physical and anthropometric measurements will be performed in the morning before breakfast. Participants will be wearing light clothes and no shoes during examination.

*Weight*: The weight scale will be placed in a hard-flat surface and properly calibrated before each measurement. All measurements will be taken with the patient standing without support on the centre of the scale with their weight distributed evenly in both foot.

*Height*: will be measured using a metric tape with the patient standing against the wall with their head in the Frankfort plane.

*Body Mass Index (BMI)*: will be calculated using the weight (kg) divided by the second power of the height (meters).

*Waist*: the circumference of the abdomen at its narrowest point will be measured using a metric tape against the skin of the participants. All measurements will be taken perpendicular to the long axis of the trunk, between the lower costal border and the top of the iliac crest.

*Heart rate*: calculated using the R-R interval in the electrocardiogram with the patient in the supine position after a 5-minute rest.

*Blood pressure (BP)*: BP will be taken using a mercury sphygmomanometer on the right arm, with the patient comfortably seated, after a 5-minute rest[42].

### Assessment of vascular function

All the assessments of vascular function will be performed in the morning, in a temperature controlled room, with participants required to fast for at least 8 hours. Flow-mediated, endothelium dependent vasodilatation of the brachial artery (FMD) will be measured by the technique described by Celermajer et al.[28] using the guidelines reported by Coretti et al.[43] The ultrasound image of the right brachial artery will be measured longitudinally 5-10 cm above the antecubital fossa by a 2D high-resolution (Siemens SG-60, USA) ultrasound device, using a 7.5 MHz linear array transducer. Ultrasound procedures will be executed with the subject resting quietly in supine position for at least 10 minutes. All measurements will be taken at end-diastole guided by the electrocardiogram. The baseline diameter of the brachial artery will be measured from the anterior to the posterior intima/lumen interface at three points located at a predetermined distance, the mean diameter of the brachial artery will be calculated as the average of the three measures. After the baseline measurement, a pneumatic tourniquet positioned around the right arm will be inflated to at least 50 mm Hg above the systolic pressure for at least five minutes. A rapid release of the cuff will induce reactive hyperemia and shear stress on the endothelium, leading to endothelium-dependent vasodilatation, mainly due to the increase of free cytosolic Ca^2+ ^concentration with subsequent activation of endothelial Nitric Oxide Synthase (eNOS) and release of NO to the vascular smooth muscle[43]. FMD will be calculated as the percentage of change in the diameter of brachial artery measured 45-60 s after cuff release in relation to the baseline measure (FMD%). After 10 minutes of rest, sublingual administration of 25 μg of nitroglycerin will be used to measure the endothelium-independent vasodilatation.

### Statistical analysis

All statistical analyses will be performed using the intention-to-treat principle unless stated otherwise. A P of 0.05 will be used as a cutoff for statistical significance. The primary outcome (difference on endothelium-dependent brachial artery FMD at baseline and three months after randomization) and the secondary outcome (hs-CRP, glucose, blood lipid profile, and HOMA index) will be analyzed using repeated measures ANOVA for continuous variables. Differences in proportions in outcomes between the treated group and the control group will tested for statistical significance by χ2-test or Fisher exact test if applicable.

### Study conduct and monitoring

This study will be conducted in accordance with accepted ethical and scientific standards (SOP) in order to protect participants, to preserve the study's scientific integrity and to identify problems and correct them efficiently. This study will also be monitored by the Human Rights Committee at the Universidad del Valle Institutional Review Board composed of experts in medicine, dentistry and clinical epidemiology. Monitoring reports will be submitted to the committee focusing on information about patient compliance, protocol adherence, data retrieval, and adverse events to the intervention. All adverse events will also be reported to the Universidad del Valle IRB. The Principal Investigators' monitoring will detect consent irregularities, unsafe confidentiality practices, and participants who are ineligible because of errors in assessing or recording relevant medical conditions in an effort to decrease adverse events. In addition, there will be weekly conference calls between the coordinating Center and researches to review procedures and address potential problems. The Coordinating Center will also ensure the quality and integrity of the data collection process.

### Ethical aspects

This clinical trial will be conducted in accordance with the Good Clinical Practice Guidelines, the Helsinki's Declaration, and the Colombian legislation as per the Resolution 8430 of 1993 from the Ministry of Health. Prior to de admission to the patients in the study, the objectives and the methodology will be explained and the patient will provide written informed consent in a form designed for such purpose. Confidentiality of the patients will be maintained in all the phases of the study. The patients may refuse to continue participating if they no longer wish to continue the study. The design and methods of this RCT are in accordance with the recently published extension of the CONSORT statement to randomized trials of non-pharmacological treatment[44].

## Abbreviations

hs-CRP: High-sensitivity C-reactive protein; ICAM-1: Intercellular adhesion molecule-1; VCAM-1: Vascular cell adhesion molecule-1; RR: relative risk; CI: confidence interval; TNF-α: Tumor necrosis factor-α; IL: Interleukin; eNOS: endothelial Nitric Oxide Synthase; MCP-1: Monocyte chemoattractant protein-1; HSP: Heat shock protein; TLR: Toll-like receptor; FMD: Flow-mediated endothelium dependent vasodilatation of brachial artery; ACE: Angiotensin converting enzyme; RCT: Randomized controlled trial; CONSORT: consolidated standards of reporting trials; HOMA: Homeostatic model assessment; SOP: Standard operating procedures; SNOSE: Sequentially numbered, opaque, sealed envelopes; GML: Gingival margin level; PD: Pocket depth; BOP: Bleeding on probing; CAL: Clinical attachment level; PCR: Polymerase chain reaction; VMGA-III: viability preserving medium no. III; EDTA: Ethylenediaminetetraacetic acid; CBC: Complete blood count; HDL-C: high-density lipoprotein cholesterol; LDL-C: Low-density lipoprotein cholesterol; TC: Total cholesterol; MMP-9: matrix metalloproteinase-9; MPO: myeloperoxidase; PAI-1: Plasminogen activator inhibitor-1; TSBV: Tryptic soy serum bacitracin vancomycin agar; HCMV: Human cytomegalovirus; BMI: Body mass index; NO: Nitric Oxide; ANOVA: Analysis of variance; IRB: Institutional review board.

## Competing interests

The authors declare that they have no competing interests.

## Authors' contributions

JR, AC, and RA contributed equally in the conception of the study, trial design and draft of the manuscript. JR drafted the protocol, AC wrote the microbiological procedures and RA was responsible for the statistical design of the trial. All authors read and approved the final manuscript.
